# Urban versus forest ecotypes are not explained by divergent reproductive selection

**DOI:** 10.1098/rspb.2018.0261

**Published:** 2018-07-11

**Authors:** Aude E. Caizergues, Arnaud Grégoire, Anne Charmantier

**Affiliations:** CEFE, CNRS, Université de Montpellier, Université Paul Valery Montpelliers, EPHE, IRD, Montpellier, France

**Keywords:** urbanization, reproductive selection, adaptation, morphology, life-history traits

## Abstract

Increasing urbanization offers a unique opportunity to study adaptive responses to rapid environmental change. Numerous studies have demonstrated phenotypic divergence between urban and rural organisms. However, comparing the direction and magnitude of natural selection between these environments has rarely been attempted. Using seven years of monitoring of great tits (*Parus major*) breeding in nest-boxes across the city of Montpellier and in a nearby oak forest, we find phenotypic divergence in four morphological and two life-history traits between urban and forest birds. We then measure reproductive selection on these traits, and compare selection between the habitats. Urban birds had significantly smaller morphological features than their rural counterparts, with a shorter tarsus, lower body mass, and smaller wing and tail lengths relative to their overall body size. While urban female tarsus length was under stabilizing selection, and forest males show positive selection for tarsus length and negative selection for body mass, selection gradients were significantly divergent between habitats only for body mass. Urban great tits also had earlier laying dates and smaller clutches. Surprisingly, we found selection for earlier laying date in the forest but not in the city. Conversely, we detected no linear selection on clutch size in the forest, but positive selection on clutch size in the urban habitat. Overall, these results do not support the hypothesis that contemporary reproductive selection explains differences in morphology and life history between urban- and forest-breeding great tits. We discuss how further experimental approaches will help confirm whether the observed divergence is maladaptive while identifying the environmental drivers behind it.

## 1. Introduction

Human activities drive tremendous environmental changes in a wide range of ecosystems around our planet. Wildlife responses to these changes provide interesting models for evolutionary biologists, as phenotypic divergence between populations is usually much more pronounced when one of the paired populations is found in a human-modified habitat [1]. Among emerging human-altered habitats, urban areas provide some of the most striking environmental changes, both in terms of the level of artificiality as well as in terms of the temporal scale of landscape changes. Cities offer a range of new environmental conditions, such as high levels of pollution, increased noise levels, artificial light, modified plant communities, new patterns of access to resources and higher temperatures [2]. Research in urban ecology during the past decade has revealed many cases of phenotypic divergence between urban and native habitats. This is especially true for birds, which have been studied for over a century [3], and for whom studies have shown divergence in morphology, physiology, behaviour, song [4–6] and life history [7], highlighting the existence of urban versus rural ecotypes (i.e. populations presenting phenotypical divergence associated with particular environmental conditions).

Although some studies have found consistent patterns across species for a few traits, such as reduced body size in urban birds [8], no general pattern of morphological divergence arises for urban populations. For instance, several studies find birds have a lower body condition in the city [5,7], and suggest that these differences might be adaptive due to higher temperatures (Bergmann's rule applied to urban habitats) and the non-necessity of fat reserves because of high resource predictability [5,9]. However, lower body mass in the city could also be maladaptive, and result from a lack of food resources (e.g. low insect abundance) [10]. Furthermore, because some studies find no difference in body mass between urban and rural birds, and others find a difference in the opposite direction [7], morphological differentiation seems to be species- and site-dependent [7,11].

The life-history ecotype of urban birds seems more generalizable than the urban morphotype (i.e. set of morphological characteristics specific to urban populations). Indeed, in the majority of species investigated, urban birds display smaller clutch sizes and advanced laying dates (reviewed in [12]). In particular, urban populations of great tits (*Parus major*) and blue tits (*Cyanistes caeruleus*) consistently have earlier and smaller clutches than those in natural habitats [13,14]. In seasonal environments, synchronization between a bird's phenology and the optimal timing of reproduction (e.g. peak in abundance of arthropods for insectivorous species) is controlled by environmental cues such as photoperiod, temperature, light and bud burst [15]. A mismatch between realized and optimal phenology can reduce individual fitness and affect population dynamics [16]. Modified environmental conditions in the city, particularly higher temperatures and novel plant communities, could lead to (1) incorrect information on the date of the optimal timing of reproduction and (2) modified access to resources. Hence, as with changes in morphology, changes in urban bird phenology could be maladaptive.

Surprisingly, the ecological and evolutionary processes leading to the phenotypic changes during the urbanization process remain largely unknown [17]. In particular, whether the new phenotypes observed in cities are adaptive and whether they are the result of ongoing micro-evolutionary processes are two of the most pressing questions in urban evolutionary ecology. The assumption that rapidly evolving urban areas offer novel selection pressures is prevalent in the literature [1,18], giving rise to a second common assumption that urban ecotypes are adaptive. In several cases, genetic studies have demonstrated that the observed phenotypic differences between urban and natural populations are genetically based (e.g. [19,20]) and have, in some cases, arisen recently despite gene flow (e.g. [21]). However, at present only a handful of studies have explicitly compared the direction and force of natural and/or sexual selection acting in urban versus natural habitats by linking phenotypes to fitness [18]. The industrial melanism in the peppered moth (*Biston betularia*) is the most famous example, demonstrating how increasing (and subsequently decreasing) air pollution induced novel selection (and subsequently reverse selection) that resulted in rapid evolution of phenotypes and genotypes associated with camouflage [22]. More recently, a study on the northern cardinal (*Cardinalis cardinalis*) revealed relaxed sexual selection on colouration for urban males compared with their forest conspecifics because of a disassociation between brightness of male plumage and territory attributes in urbanized areas [23].

The present study addresses the fundamental question: does selection differ in direction and magnitude between a forest and an urban avian population? Our work is divided into two parts. First, we analyse phenotypic divergence between forest and urban great tits for several morphological and life-history traits, using a long-term study in and near the city of Montpellier, France. Second, we perform selection analyses on these traits and compare patterns of reproductive selection between forest and urban habitats. Based on previous studies on other populations of the same species, we predict that urban great tits will present smaller morphological characteristics than their forest counterparts. We also predict an earlier laying date in the city due to higher temperature perceived as an indicator of earlier spring and smaller clutch size linked to fewer food resources. We then test whether the divergences found in morphology and life history are aligned with differences in the direction and/or force of reproductive selection.

## 2. Material and methods

### (a) Study areas, monitoring and phenotyping

Two populations of great tits (*P. major*) were monitored in the south of France: one in a forested area and one in an urban area. The forested site, in the forest of La Rouvière, is situated 20 km northwest of the city of Montpellier. At this site, between 51 and 92 great tit nest-boxes have been monitored since 1991 [24]. The urban site is within the city of Montpellier, and contains 203–223 nest-boxes that have been monitored since 2011, providing data on great tits across various levels of urbanization [13,25]. The forest and urban sites presented largely dissimilar environmental conditions. In the city, nest-boxes were not only placed in parks, but also on trees near pavements, roads and street lamps, thus exposed to human disturbance, air and light pollution. Moreover, the vertical matrix is highly modified with numerous buildings and reduced vegetation cover composed of many ornamental flowers and trees such as platanus (*Platanus* × *acerifolia*), olive trees (*Olivacea europea*) or resinous trees. By contrast, the forest site was dominated by downy oaks (*Quercus pubescens*) and holm oaks (*Quercus ilex*), with limited human disturbance and pollution levels. Hence the rural site corresponds to a mature stage of a Mediterranean forest succession, while the urban site corresponds to a human-dominated place with a higher proportion of artificial cover. On average, nest-box environment (estimated based on satellite image analysis within a 100 m diameter circle around each nest-box) was covered at 61.9% by vegetation in the city versus 98.9% in the forest, the remaining percentage being impervious surfaces. Satellite pictures of both sites and details on estimation in green cover are available in the electronic supplementary material (figure S1 and appendix S1).

Nest-boxes were visited at least once a week during the breeding season to follow brood development. Parents were captured inside nest-boxes when their nestlings were between 9 and 15 days old and were individually measured for several morphological (i.e. tarsus length, body mass, wing and tail lengths) and behavioural traits, and ringed with unique metal rings. For more details about the monitoring protocol see electronic supplementary material, appendix S1. At the urban site, although brood development traits have been monitored since 2011, parents have only been captured since 2013. In the analysis, only years for which both study sites were sampled are considered. Between 2011 and 2017, a total of 192 forest broods and 546 urban ones were monitored for which 153 forest and 391 urban birds were captured (from once to four times between 2013 and 2017).

### (b) Statistical analyses

All data analyses were performed using the software R (version 3.4.0).

#### (i) Urban versus forest phenotypic divergence

Differences between the two habitats in great tit morphology were explored using linear mixed models (packages *lme4* and *lmerTest*) on four traits: tarsus length, body mass, wing length and tail length. Full models included year (as a categorical variable), habitat (urban versus forest), sex (male versus female) and age (yearling versus adult) as fixed effects, with the following interactions: habitat × age, habitat × sex, habitat × year, sex × age, sex × year. Individual ring number and measurer identity were fitted in all models as random effects. As morphological traits were highly correlated (see all between-trait correlations in electronic supplementary material, table S1), measures of body mass, wing length and tail length were scaled for the general bird size by including tarsus length as a covariate, thereby modelling body condition and relative wing and tail lengths. Avian tarsus length is a common index of structural size of the whole bird (e.g. [26]). As great tit body mass is known to vary consistently during the day in a linear manner (e.g. [27]), the hour of measure was added as a continuous explanatory variable in body mass models.

To test for differences in laying date and clutch size (hereafter referred to as LD and CS, respectively) between the city and the forest, we considered only first broods because second and replacement broods were rare and would require a separate analysis. As above, linear mixed models were run with habitat, year and habitat × year as fixed effects. LD was added in the CS model to account for the fact that late females usually lay smaller clutches [13,28]. Female identity was included as a random effect to account for the non-independence of broods from the same females. Because the monitoring did not include parental captures during the first two years and because all females were not captured (when abandoning before chicks reached 9 days old, death or failure to capture), female identity was unknown for 54% of broods (342 out of 739). To avoid the loss of half of our dataset, we attributed a fictitious ring number to each non-captured female.

For each trait, the best model was selected using a backward stepwise procedure starting from a full model, and the significance of each fixed effect and interaction was tested using a *F*-test (R function anova()). This procedure was used to eliminate non-significant variables: at each step, the effect presenting the highest *p*-value was removed and the model run again without it. While we present only ANOVA of the best-fitting models below, summaries of full and best-fitting models are provided for each trait in the electronic supplementary material (tables S2–S5).

#### (ii) Measuring and comparing the force of selection

We estimated reproductive selection for the focal morphological and life-history traits, and subsequently tested whether the direction and force of selection varied between the urban and forest habitats. Separate analyses were run for each sex because of sexual dimorphism (males are larger, e.g. [29] and table 1).

**Table 1.** Comparing the morphological traits tarsus length (mm), relative body mass (g), relative wing length (mm) and relative tail length (mm) between forest and urban great tits using linear mixed models. Habitat-specific means (raw data) and output of *F*-tests for significant fixed effects (in bold). *N*_tarsus_ = 554, *N*_mass_ = 550, *N*_wing_ = 553 and *N*_tail_ = 537.

mean ± s.d.	tarsus length				body mass				wing length				tail length			
	forest		city		forest		city		forest		city		forest		city	
females	19.54 ± 0.51		19.23 ± 0.53		16.61 ± 0.88		16.20 ± 0.89		73.39 ± 1.80		72.58 ± 1.70		61.24 ± 2.55		60.87 ± 2.43	
males	20.01 ± 0.51		19.76 ± 0.57		17.25 ± 0.94		16.98 ± 0.97		76.43 ± 2.09		75.59 ± 2.29		65.35 ± 2.84		64.37 ± 2.93	
fixed effects	sum sq.	d.f.	*F*	***p***	sum sq.	d.f.	*F*	***p***	sum sq.	d.f.	*F*	*p*	sum sq.	d.f.	*f*	*p*
habitat	0.54	1	22.35	**3 × 10^−6^**	2.88	1	9.57	**0.002**	46.86	1	17.52	**7 × 10^−5^**	39.44	1	12.25	**0.001**
year	n.s.	n.s.	n.s.	n.s.	24.22	4	20.15	**4 × 10^−15^**	46.69	4	4.37	**0.002**	65.78	4	5.11	**0.001**
sex	2.30	1	94.66	**<2 × 10^−16^**	6.30	1	20.96	**6 × 10^−6^**	455.90	1	170.47	**<2 × 10^−16^**	510.34	1	158.48	**<2 × 10^−16^**
age	n.s.	n.s.	n.s.	n.s.	6.02	1	20.04	**9 × 10^−6^**	251.82	1	94.16	**<2 × 10^−16^**	236.93	1	73.58	**<2 × 10^−16^**
tarsus length	—	—	—	—	32.33	1	107.56	**<2 × 10^−16^**	104.73	1	39.16	**1 × 10^−9^**	14.10	1	4.38	**0.037**
habitat×year	n.s.	n.s.	n.s.	n.s.	2.93	4	2.43	**0.047**	30.96	4	2.89	**0.023**	n.s.	n.s.	n.s.	n.s.
sex×age	n.s.	n.s.	n.s.	n.s.	n.s.	n.s.	n.s.	n.s.	16.90	1	6.32	**0.012**	n.s.	n.s.	n.s.	n.s.
hour	—	—	—	—	2.64	1	8.77	**0.003**	—	—	—	—	—	—	—	—

The choice of an optimal estimation of relative fitness to adequately measure natural and sexual selection is a long-standing debate [30] with no straightforward solution. As our goal was to understand the adaptive mechanisms explaining urban/forest divergence in a set of traits with little access to complete life histories, we opted for an annual measure of fitness rather than lifetime reproductive success [31]. Also, because of the short time scale of our dataset and following arguments that natural selection is a within-generation process [32], we chose the number of fledglings as a fitness measure rather than the number of recruits.

We first estimated standardized univariate selection differentials (*S*) on all traits individually [33,34] in each habitat separately. The linear selection differential for a trait is estimated by the slope of the regression between a measure of relative fitness and standardized values of the phenotypic trait. Selection differentials measure the total force of selection affecting a trait, including both direct and indirect selection [32]. As all four morphological traits were correlated with body size (electronic supplementary material, table S1), estimations of selection differentials were performed on measures corrected for size by using residuals of a linear regression between the trait (body mass, wing or tail length) and tarsus length run on the pooled dataset with both urban and forest birds. For body mass, the time at which each measurement was taken was added as an additional explanatory variable in the linear regression, thereby providing a measure of body condition corrected for time of day.

To identify the target of selection when multiple, potentially correlated, traits are simultaneously studied, multivariate selection gradients *β* [32] estimate direct selection on each trait after removing indirect selection from other traits. These multivariate models were run for normally standardized morphological and life-history traits separately. For example, linear selection gradients *β*_i_ on morphological traits were estimated by the model2.1
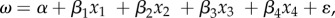
where *ω* is the relative fitness, *α* is the intercept, *x*_1_ is the tarsus length, *x*_2_ is the body mass, *x*_3_ is the wing length, *x*_4_ is the tail length and *ɛ* is the error. We also estimated nonlinear quadratic (*γi*) and correlational (*γ_ij_*) selection gradients from the following model [35]:2.2
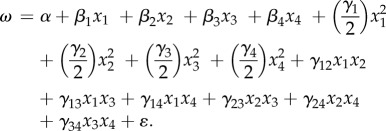


When estimating selection gradients, correction for tarsus length was not needed because tarsus length was already in the model (equations (2.1) and (2.2)). Hence, only body mass was corrected for hour of the day using residuals from a linear regression between body mass and hour of measure. Selection gradients on life-history traits in each habitat were estimated in the same way, with LD and CS as traits 1 and 2 in (2.1) and (2.2).

Estimation of parameters and tests of significance were conducted in two steps (e.g. [36]). First, estimates of selection differentials and gradients were obtained from linear models as described by equations (2,1) and (2.2). Second, significance (i.e. *p*-values) of these selection differentials and gradients were estimated from linear mixed models including year and individual ring number (only female identity for life-history traits) as random effects to control for variation across years and repeated measurements on the same individuals. Then, to test for a difference in selection operating in the two habitats, urban and forest datasets were combined and habitat (forest versus urban) and habitat × trait were added as fixed effects in the models described above. Significance of the interaction terms was tested using *F*-tests (R function anova()).

Finally, because variance can provide insight on past selection as well as relaxed selection, a comparison of variance was made between habitats for each trait with a Fisher variance ratio test (var.test(), R package *stats*).

## 3. Results

### (a) Urban versus forest differences in morphology and life history

**Figure 1.**
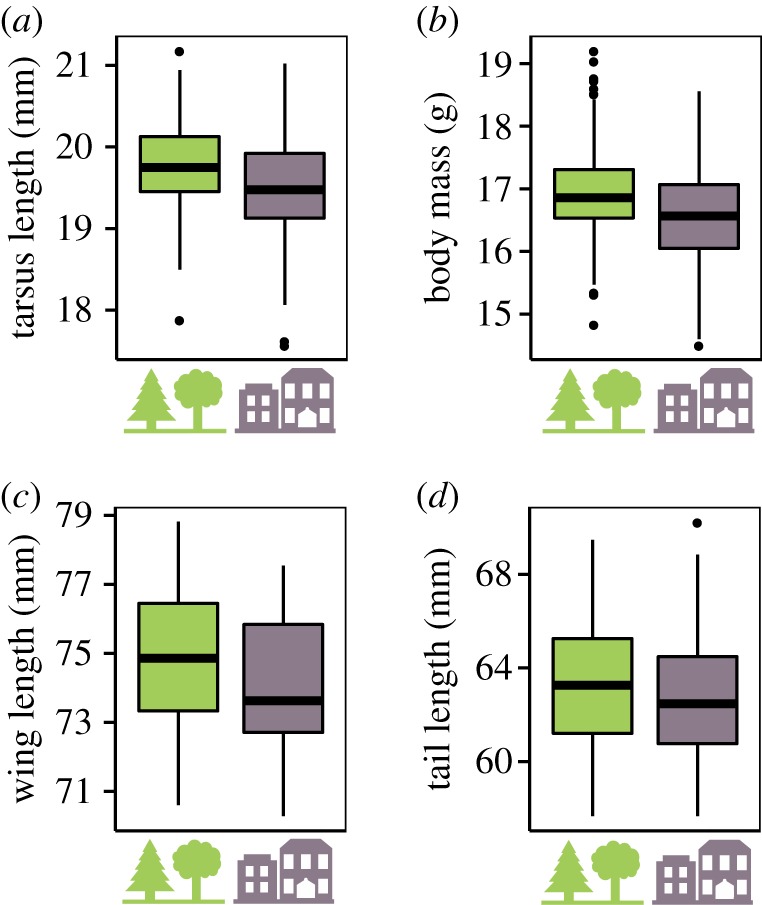
Morphological divergence between forest (green) and urban (purple) great tits for (*a*) tarsus length, (*b*) body mass, (*c*) wing length and (*d*) tail length. Boxplots of predicted data from the best linear mixed models, representing minimum, first quartile, median, third quartile, maximum and outliers (points).

Comparison of morphological features between urban and forest great tits revealed a strong phenotypic differentiation (figure 1). Indeed, for all four traits, habitat (urban versus forest) had a significant effect either alone or in an interaction (table 1). First, urban birds were smaller than their forest counterparts, as shown by a tarsus length 1.3 to 1.6% smaller (males and females) in urban birds (*p* < 0.001; table 1; electronic supplementary material, table S3). Second, urban birds had lower body mass corrected for tarsus length (interaction = 0.002; table 1; electronic supplementary material, table S3). Third, urban birds displayed shorter wings and tails (*p* < 0.001 and *p* = 0.001; table 1; electronic supplementary material, table S3). In both populations, there was a significant sexual dimorphism, with males larger than females (table 1). We also noted an effect of year, either directly or via an interaction, for all morphological traits but tarsus length (see electronic supplementary material, figures S2–S4). For instance, differences in wing length between the habitats varied between years (significant habitat × year interaction, *p* = 0.023; table 1; electronic supplementary material, table S3). Moreover, yearlings displayed smaller features than adults (except for tarsus length, which is fully grown at 15 days of age; electronic supplementary material, table S3).

**Figure 2.**
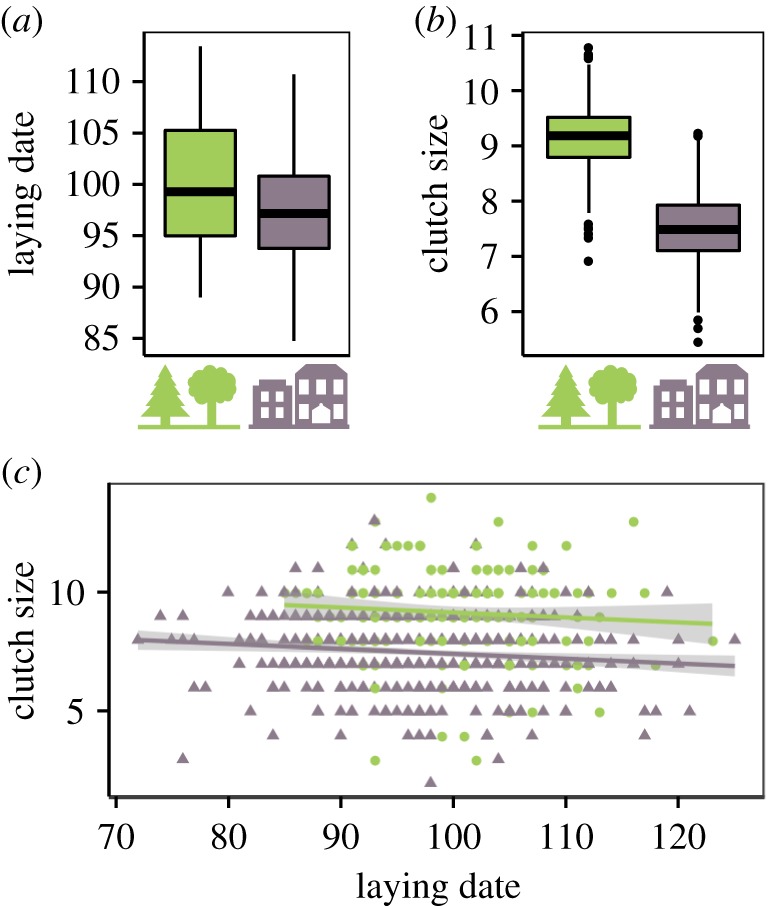
Divergence in (*a*) laying date and (*b*) clutch size between forest (green) and urban (purple) great tits. Boxplots of predicted data from the best linear mixed models, representing minimum, first quartile, median, third quartile, maximum and outliers (points). (*c*) Correlations between laying date and clutch size in both habitats (raw data) with 95% confidence intervals in grey.

Concerning life-history traits, urban females laid eggs on average 4.05 days before forest females (*p* = 0.032; table 2; figure 2; electronic supplementary material, table S5; figure 2) and their earliness showed annual fluctuations (significant habitat × year interaction, *p* = 0.02; table 2; electronic supplementary material, table S5). Mean LD varied annually with a magnitude of 9 days across the 7 years of monitoring. Urban females laid 1.75 less eggs on average than their forest counterparts (*p* < 0.001; table 2; figure 2; electronic supplementary material, table S5). As expected, we observed a negative correlation between LD and CS when accounting for habitat and year as fixed effects and female identity as a random effect (*p* = 0.03; table 2; figure 2).

**Table 2.** Comparing laying date and clutch size between forest and urban great tits using linear mixed models. Habitat-specific means (raw data) and output of *F*-tests for significant fixed effects (in bold). *N*_urban_ = 546 and *N*_forest_ = 192.

	laying date				clutch size			
	forest	city			forest	city		
mean ± s.d.	100.01 ± 7.24^a^	97.2 ± 8.35^a^			9.16 ± 1.75	7.46 ± 1.51		
fixed effects	sum sq.	d.f.	*F*	***p***	sum sq.	d.f.	*F*	***p***
habitat	1033.4	1	36.18	**3 × 10^−9^**	261.35	1	157.63	**<2 × 10^−16^**
year	7521.5	6	43.88	**<2 × 10^−16^**	34.68	6	3.49	**2 × 10^−3^**
habitat × year	444.9	6	2.60	**0.02**	5.97	6	0.60	0.73
laying date	—	—	—	—	7.70	1	4.65	**0.03**

^a^Laying date is expressed in ordinal date, where 1 = 1 January.

### (b) Selection on morphology

Only selection gradients are presented in the main results (see electronic supplementary material, table S6 and figure S5 for selection differentials). Regarding females, no linear selection was detected on morphological traits, but we found stabilizing selection on tarsus length in the city (*γ* = −0.166, *p* = 0.036; electronic supplementary material, table S7). However, pooling the urban and forest datasets revealed that this quadratic selection was not significantly different between habitats (non-significant tarsus^2^ × habitat interaction, *p* = 0.632; electronic supplementary material, table S7). Concerning males, we detected linear selection for larger tarsi in the forest (*p* = 0.01; table 3), but once again the difference in selection between habitats was non-significant (non-significant tarsus × habitat interaction, *p* = 0.275; table 3). Moreover, we detected significant negative linear selection on body mass for forest males (table 3), and a significant difference in selection between city and forest (significant body mass × habitat interaction, *p* = 0.034; table 3). This result suggests that lighter birds have higher reproductive success in the oak forest but not in the urban habitat. Finally, no difference in variance was found in any morphological trait (electronic supplementary material, table S8).

**Table 3.** Standardized linear selection gradients, estimated from multivariate analyses on tarsus length (*β*_tarsus_), body mass (*β*_body mass_), wing length (*β*_wing_) and tail length (*β*_tail_). Values are provided with their standard error. Bold estimates are significant (*p* **<** 0.05). ‘Interaction' displays the value of the interaction between each trait and habitat in the merged (forest + city) model.

sex	habitat	sample size	linear selection gradients							
			*β*_tarsus_		*β*_body mass_		*β*_wing_		*β*_tail_	
			est. ± s.e.	interaction	est. ± s.e.	interaction	est. ± s.e.	interaction	est. ± s.e.	interaction
females	forest	104	0.037 ± 0.068	−0.037	−0.050 ± 0.066	−0.025	0.029 ± 0.068	0.025	−0.096 ± 0.068	0.062
	city	200	−0.032 ± 0.049		−0.046 ± 0.049		0.098 ± 0.052		−0.027 ± 0.049	
males	forest	94	**0.138 ± 0.067**	−0.085	−**0.179 ± 0.068**	**0.167**	−0.004 ± 0.076	0.057	0.062 ± 0.075	0.016
	city	191	−0.018 ± 0.043		0.050 ± 0.056		0.025 ± 0.066		0.0004 ± 0.060	

### (c) Selection on life-history traits

As above, the selection differentials and gradients show similar patterns, and we, therefore, present only the results for selection gradients (but see electronic supplementary material, tables S9 and S10 for selection differentials and quadratic selection gradients on LD and CS). There was negative linear selection favouring earlier breeding in the forest (*p* = 0.031; table 4). In contrast, LD was under no selection in the city (table 4; electronic supplementary material, figure S5). In the model combining both habitats, the LD × habitat interaction was significant (*p* < 0.001; table 4), with a difference of 0.31 between the two standardized gradients. A comparison of variances showed higher variance in LD at the urban site (electronic supplementary material, table S8), suggesting relaxed selection on LD in the city.

**Table 4.** Standardized linear selection gradients estimated for laying date (*β*_LD_) and clutch size (*β*_CS_). Values are provided with their standard error. Bold estimates are significant (*p* < 0.05). ‘Interaction' displays the value of the interaction between each trait and habitat in the merged (forest + city) model.

habitat	sample size	linear selection gradients			
		*β*_LD_		*β*_CS_	
		est. ± s.e.	interaction	est. ± s.e	interaction
forest	185	−**0.14 ± 0.06**	**0.31**	0.08 ± 0.06	0.12
urban	524	0.09 ± 0.03		**0.21 ± 0.03**	

The force of selection on CS also differs between the two habitats (electronic supplementary material, figure S5): great tits showed significant positive linear selection for CS in the city (*p* < 0.001; table 4), but not in the forest (table 4). The pooled model revealed that this difference in selection affecting urban and forest CS was marginally significant (CS × habitat interaction, *p* = 0.054; table 4). Finally, variance in CS was higher in the forest than the city (electronic supplementary material, table S8).

## 4. Discussion

In this study, we investigated whether an urban ecotype existed in great tits breeding in the city of Montpellier, and whether this ecotype could be explained by divergent selection between urban and forest habitats. We highlighted the existence of an urban morphotype in great tits, whereby urban birds showed smaller body size, reduced body mass, and smaller wing and tail lengths, compared with their forest counterparts. Moreover, urban great tits displayed earlier breeding phenology and smaller clutches. Selection analyses showed no differences in selection on morphological features between habitats, except for body mass in males, whereby lighter males had higher reproductive success in the forest but not in the city. On the contrary, life-history traits displayed strong selection gradients, with an interesting contrasted selection between the city and the forest. Patterns of selection on laying date (LD) varied between habitats in both direction and strength: while early females were favoured in the forest, there was non-significant positive selection on LD in the city. Finally, selection on clutch size (CS) differed in strength between the city and the forest, with significant selection favouring large clutches in the city but non-significant selection in the forest. We discuss below how this direct comparison of the force of selection operating in urban and forest habitats improves our understanding of evolution in cities.

### (a) Is morphological divergence adaptive?

Our morphotype analysis is, in part, consistent with previous studies on urban birds. Most passerine studies on nestlings or reproductive individuals have reported that urban birds are smaller than their counterparts from native environments [4,5,37]. On the contrary, the finding that birds in the city had a lower body mass than those in the forest is consistent with some studies (e.g. [5]), but not all [4,37]. Several city features have led to predictions that being smaller could be associated with a stronger fitness advantage in an urban habitat than in a forest habitat. First, cities are ‘urban heat islands’ [2]. Hence, following Bergmann's rule [38], warmer city environments should translate into a smaller adaptive body size. Second, the ‘credit card hypothesis' [39] postulates that higher food resource predictability in cities should allow urban birds to live without accumulating fat reserves. However, our selection analysis did not support the prediction of stronger negative directional selection in the city. If anything, the reverse could be true, because the only significant difference in selection gradient for morphological traits revealed that leaner males had higher reproductive success in the forest, but not in the city (table 3). However, such a negative selection gradient on body mass is not unusual [40] and should be interpreted with care. As body mass was measured for parents during the nestling feeding period, it is highly likely that the association between the number of fledglings and paternal body mass results from active males with large broods losing more weight during the reproductive period. It remains to be explained why urban fathers would not lose weight compared with forest fathers during the reproductive season. Overall, these results suggest that the morphological differences between urban and forest birds are not an adaptive response to divergent selection operating presently. Note that the absence of significant selection on male and female morphology in urban birds could also be indicative of a fast evolution towards the urban morphotype following strong past selection. The hypothesis of an evolutionary stable optimum reached in the city environment is, however, not supported by (a) a variance comparison (electronic supplementary material, table S8) revealing no difference between habitats for all morphological trait variances, and (b) the documented population genetic structure. Indeed, a recent genomic study showed low genetic differentiation between the two focal populations (*F*_ST_ close to 0.01) [41], suggesting strong gene flow, and hence small potential for local adaptation [42].

Our finding of smaller relative wing and tail length in urban great tits is comparable with only one published study, on the European blackbird *Turdus merula*, where a comparative approach across 11 paired urban and rural sites [11] found inconsistent results across sites, but generally longer wings in urban birds. Wing and tail morphologies are directly linked to flight aerodynamics, for example, shorter wings and longer tails increase manoeuvrability [43]. The urban habitat being substantially different in its vertical matrix (e.g. buildings) compared with a forest, and having an increased collision risk (e.g. from numerous vehicles), it is easy to imagine that modified plumage characteristics could be advantageous. However, our selection analyses for relative wing and tail length show no evidence for reproductive selection on either trait in either habitat.

Overall, while the weak selection on morphology is consistent with previous studies in passerines [40,44], our analyses provide no support for an adaptive explanation for the morphological divergence between city and forest great tits. Thereby, morphological plasticity remains the most likely process explaining the observed phenotypic differentiation, and could be a maladaptive product of unfavourable environmental conditions [5]. This hypothesis remains to be tested using common garden or cross-fostering protocols. Such experimental approaches would help identify which environmental features (such as food abundance and predictability, predation pressure, pollution) are responsible for the divergent morphotypes. Also, while our selection analysis focuses on the breeding period, we are well aware that selection can vary among life stages, thus calling for studies that consider cumulative effects of selection over the life cycle. A complementary analysis of selection via survival is necessary to reach conclusions regarding the overall selection pressures affecting the focal traits in the two habitats. Developing a unique fitness measure that combines survival and reproduction could provide a different picture of the adaptive nature of the morphological divergence observed here [45].

### (b) Maladaptive shift in urban life history

The observed differences in life-history traits, with an earlier LD and smaller CS in the city than in the forest, are consistent with previous findings [12]. The advanced LD could be explained by modified environmental conditions in the city. Indeed, passerines follow environmental cues to time their egg laying, particularly temperature [46]. For instance, small temperature differences inside nest-boxes influence LD [47]. Also, artificial light and differences in food resource availability could advance the phenology of urban birds [12]. Smaller CS in the city could be adaptive because of the restriction in food resources for nestlings [12] or because of higher brood predation risk [48]. Once again, these arguments have, to our knowledge, not been associated with attempts to compare selection in urban and rural environments. Here, we provide the first demonstration of a difference in reproductive selection between the two habitats. While there is reproductive selection for early LD in the forest as is often observed in rural great tit populations [49], no selection is detected in the city. This difference in selection could be explained (a) by traditional environmental cues for optimal LD timing becoming unreliable in the urban habitat, and/or (b) by relaxed selection due to anthropogenic disturbances changing selective regimes [50]. In particular, females usually match their phenology to a peak in caterpillar abundance using environmental cues, yet there might be no such peak in the city [51], hence selection for LD might be relaxed, as suggested by higher variance in urban LD. Exploring the role of food abundance and timing would require fine-scale entomological data in our urban sites to evaluate the composition and abundance in urban arthropods and the link between arthropod phenology and environmental cues. It has been demonstrated that more favourable pre-laying feeding conditions, induced by anthropogenic food supplementation during winter, often advance avian LD [12]. As food supplementation has no link with natural food availability and cannot contribute to nestling diet, such human-induced early LD might not be advantageous [12]. Likewise, because we found selection favouring large clutches in urban birds, the observed smaller and less variable CS probably results from a constraint emerging from poor food resources in the urban habitat [52,53].

### (c) Limitations and perspectives

Our analyses provide a first step towards understanding the way that natural selection shapes avian urban wildlife, but we are well aware that complementary analyses will be necessary before reaching any final conclusion about the selection patterns in the two habitats. In particular, our study focuses on a single pair of populations, and therefore we must be cautious about drawing general inferences [54]. The good news is that the abundance of cities sets an ideal opportunity to explore selection gradients in rural/urban pairs of populations in a replicated fashion, and to identify which selection drivers differ between these two types of habitats. We particularly encourage urban evolutionary scientists working on great tits to reproduce our study in their population(s) to allow a comparative approach across the species' distribution. Targeting recent colonization of urban environments would be optimal to estimate selection in great tit populations experiencing the city as a novel breeding habitat.

The high demands of gathering long-term data on individual phenotypes and fitness probably explain why, three decades after the seminal work of Lande & Arnold [32], very few analyses have compared the force and direction of natural selection between city and native habitats. New genomic tools have offered the prospect of studying the signatures of urban adaptation without the necessity to conduct long-term studies [18], with the added advantage of exploring candidate genes for specific traits involved in urban adaptation. For instance, behavioural differences observed between urban and rural blackbirds were related to divergence in the SERT gene, involved in exploratory behaviour [55]. However, in cases where urban organisms display new phenotypes as part of a plastic response, such genomic approaches will not be useful to understand whether this plasticity is adaptive. Direct estimation of selection gradients and genomic investigations are therefore best seen as complementary and should ideally be paired.

## Supplementary Material

Electronic Supplementary Materials

## Data accessibility

Dryad (http://dx.doi.org/10.5061/dryad.tv45802) [56].
