# Supplementary material for: Urban versus forest ecotypes are not explained by divergent reproductive selection
Source: Proc Biol Sci. 2018 Jul 11;285(1882):20180261. doi: 10.1098/rspb.2018.0261 (PMC6053928; doi:10.1098/rspb.2018.0261)
Supplement: Electronic Supplementary Materials [file rspb20180261supp1.pdf]

Electronic Supplementary Materials

Urban *versus* forest ecotypes are not explained by divergent reproductive selection

Aude Emilie Caizergues, Arnaud Grégoire, Anne Charmantier

Proceedings of the Royal Society B, DOI: 10.1098/rspb.2018.0261

Appendix S1: Details on the field sites and monitoring protocol 2

Figure S1: Satellite images of the study sites. 3

Figure S2: Variation in great tit body mass across years and between habitats 4

Figure S3: Variation in great tit wing length across years and between habitats 5

Figure S4: Variation in great tit tail length across years and between habitats 6

Figure S5: Distribution of linear selection gradients. 7

Table S1: Pearson correlation coefficients between morphological traits and between laying date and clutch size 8

Table S2: Output of complete initial linear models (LMER) on morphological traits 9

Table S3: Comparing the morphological traits tarsus length, relative body mass, relative wing length and relative tail length between forest and urban great tits using linear mixed models 10

Table S4: Output of complete initial linear models (LMER) on life-history traits 11

Table S5: Comparing laying date and clutch size between forest and urban great tits using linear mixed models 12

Table S6: Standardised linear and quadratic selection differentials on tarsus length, body condition, wing length and tail length. 13

Table S7: Standardised quadratic selection gradients on tarsus length, body mass, wing length and tail length. 14

Table S8: Comparison of variances of morphological and life history traits between urban and forest habitat. 15

Table S9: Standardised linear and quadratic selection differentials on laying date and clutch size..... 16

Table S10: Standardised quadratic selection gradients on laying date and clutch size. 17

Supplementary references: 18

Appendix S1: Details on the field sites and monitoring protocol

Monitoring of great tit populations took place in the city of Montpellier, France (43°36’N, 3°52’E) and in the forest of la Rouvière near Montarnaud, France (43°40’N, 3°40’E). Montpellier is the seventh most populated city in France with a total of 277 639 inhabitants in 2015 and a density of 4 881 inhabitants per square kilometre. Its urban area spreads over 57 square kilometres with 11 square kilometres of green spaces. In autumn 2010, 283 nest-boxes were placed across the city, but at the beginning of the breading season in 2011, 74 of them had already been stolen or vandalised. Since then, depending on our replacement effort and number of thefts, the number of nest-boxes fluctuated between 203 and 223 among years. Nest-boxes with 32 mm entrance holes were nailed at 2m height or higher, on a variety of trees such as: holm and downy oaks (*Quercus ilex* and *Q. pubescens*), platanus (*Platanus* × *acerifolia*), European nettle tree (*Celtis australis*), black locust (*Robinia pseudoacacia*), pines (*Pinus halepensis*, *P. pinaster* and *P. pinea*) and olive trees (*Olea europea*).

The forest of La Rouvière is a historical study site where blue tit and great tit populations have been monitored since 1991, with between 51 and 92 great tit nest-boxes placed in cages hanged on top of posts to avoid nest predation. On both sites, great tit nest-boxes were spaced by 100m to avoid intraspecific competition.

Great tits are known to explore an area of 50m radius around their nest-box (Naef-Daenzer 2000). Hence, proportions of green cover were measured for each nest-box in a circle of 100m diameter around the nest-box, giving a measure of habitat artificialisation level. Measures of green cover (*versus* impervious surfaces) around each nest-box were obtained by analysing satellite pictures with QGIS (2018). In the forest, Great tit territories were on average covered at 98.9±2.3% (mean±SD) by vegetation whereas this proportion droped to 61.9±26.9% in the city, the remaining 38.1% being artificial surfaces (roads, sidewalks, buildings).

Each week, all nest-boxes were visited to follow nest and brood development, providing information on laying date, clutch size, incubation period, number of hatchlings, age of chicks and number of fledglings. Parents were captured inside nest-boxes with mechanical traps when chicks were at least 9 days old, measured for morphological and behavioural traits and ringed with unique metal rings provided by the CRBPO (Museum National d’Histoire Naturelle, Paris). In particular, we measured body mass to the nearest 0.1g with a Pesola spring scale, tarsus length to the nearest 0.01mm with a digital calliper, and wing length and tail length to the nearest 0.5mm with rulers. All these morphological measures, apart from tail length which is rarely measured, have been shown highly repeatable and heritable (Gosler & Harper 2000 & Postma 2014).

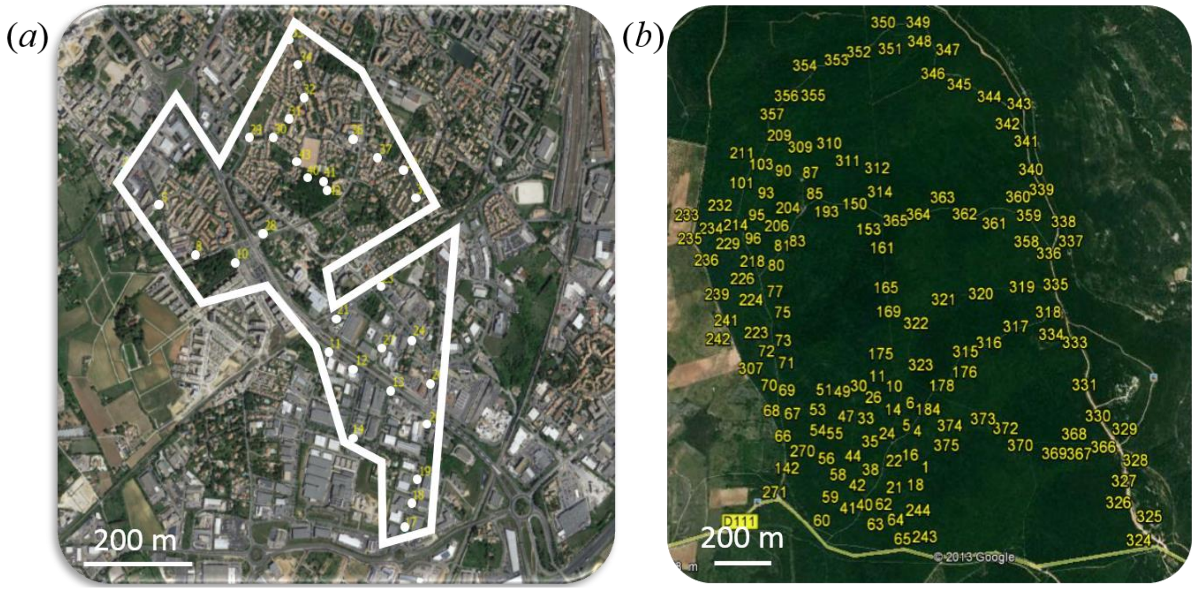

Figure S1: Satellite images of the study sites. (a) One section of the urban city site of Montpellier, France. Each dot represents a nest-box. (b) The forest site, in the forest of La Rouvière, Montarnaud, France. Each number represents a nest-box. Note that for the forest site, numerous nest-boxes are designed for blue tits (entrance whole of 28 mm). © Google Earth.

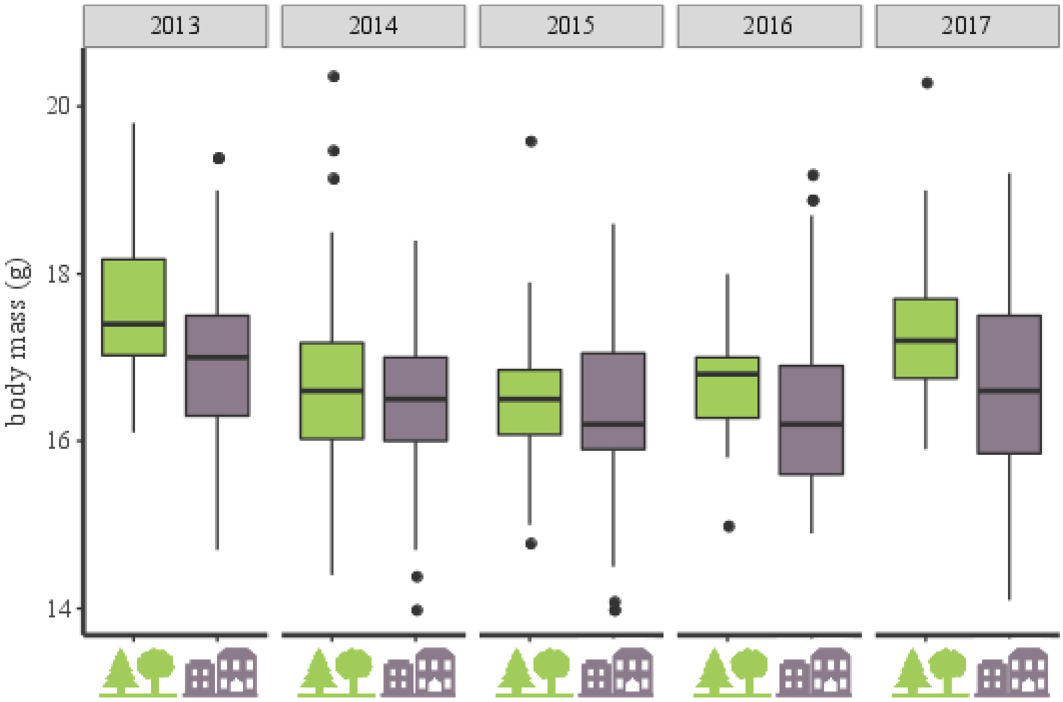

Figure S2: Variation in great tit body mass across years and between habitats (green = forest, purple = urban). Boxplots of predicted data from the best linear mixed model, representing minimum, 1st quartile, median, 3rd quartile and maximum.

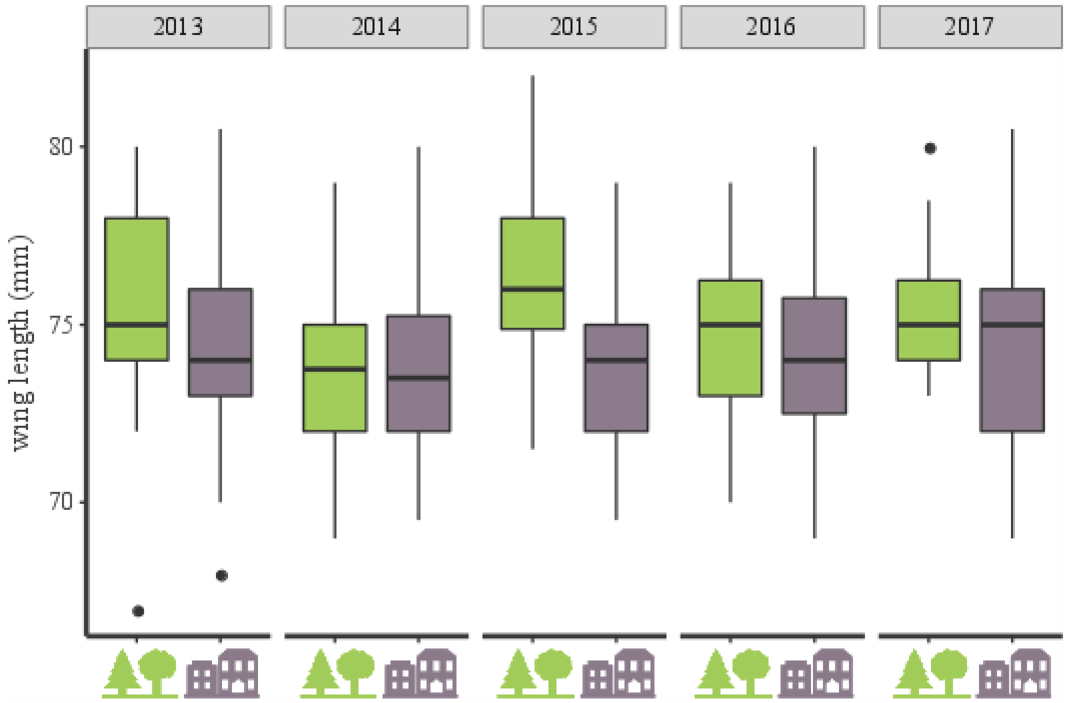

Figure S3: Variation in great tit wing length across years and between habitats (green = forest, purple = urban). Boxplots of predicted data from the best linear mixed model, representing minimum, 1st quartile, median, 3rd quartile and maximum.

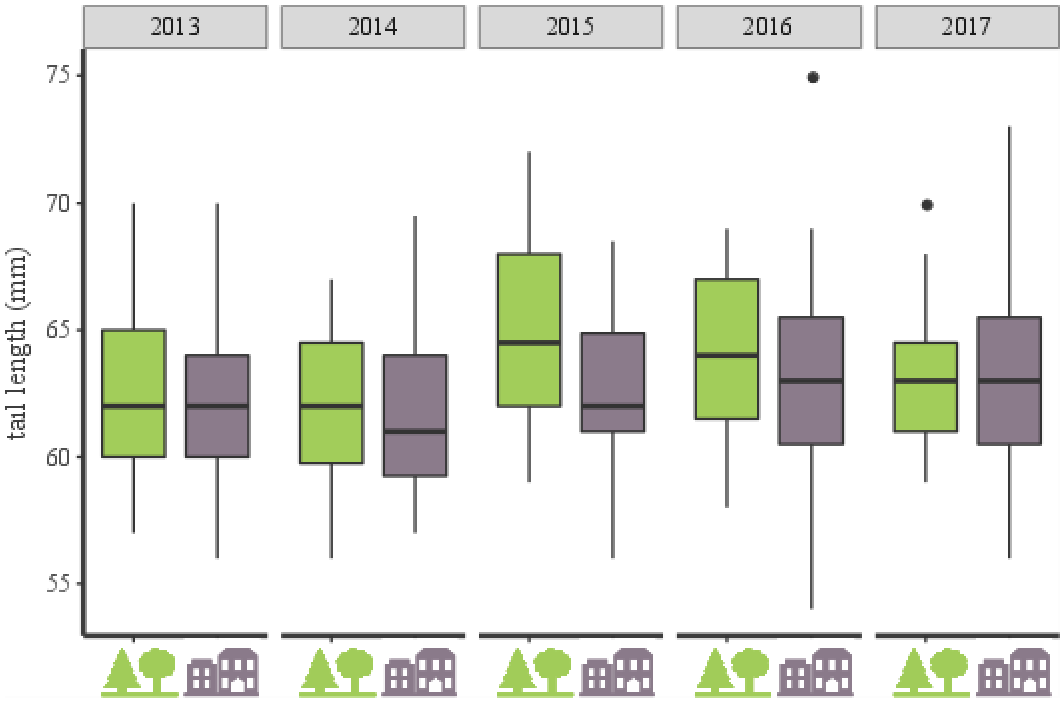

Figure S4: Variation in great tit tail length across years and between habitats (green = forest, purple = urban). Boxplots of predicted data from the best linear mixed model, representing minimum, 1st quartile, median, 3rd quartile and maximum.

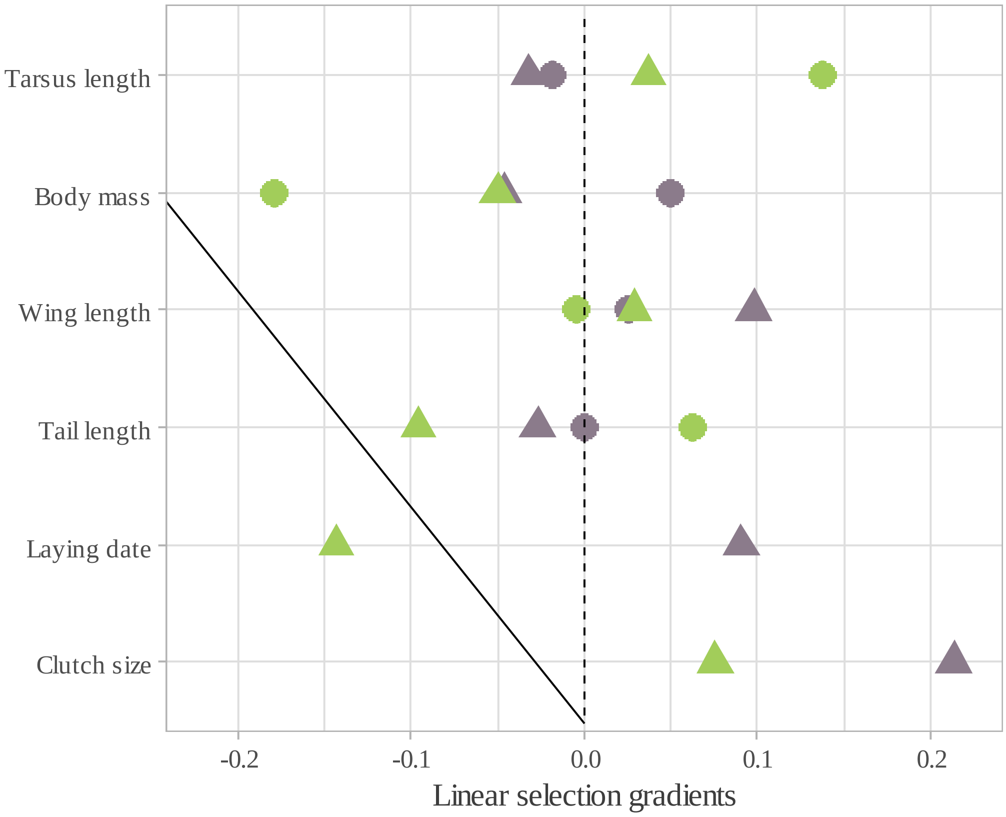

Figure S5: Distribution of linear selection gradients. Green symbols represent selection in the forest habitat and purple symbols selection in the urban habitat. Triangles represent females, circles males.

Table S1: Pearson correlation coefficients between morphological traits and between laying date and clutch size (R function cor.test). Number of observations for morphological traits N_{forest}=245 and N_{city}=517 and for life-history traits N_{forest}=192 and N_{city}=543.

trait 1	trait 2	forest		city	
		correlation	*P*	correlation	*P*
tarsus length	body mass	0.387	5.84.E-10	0.531	<2.20.E-16
tarsus length	wing length	0.33	1.30.E-7	0.467	<2.20.E-16
tarsus length	tail length	0.296	1.61.E-5	0.371	<2.20.E-16
body mass	wing length	0.345	8.33.E-14	0.533	<2.20.E-16
body mass	tail length	0.238	6.25.E-4	0.469	<2.20.E-16
wing length	tail length	0.662	2.20.E-16	0.715	<2.20.E-16
laying date	clutch size	-0.085	0.25	-0.114	0.009

Table S2: Output of complete initial linear models (LMER) for morphological traits before removing non-significant variables. Abbreviations: fem = female, hbt = habitat, yrl = yearling, nb indiv. = number of individuals, nb obs. = number of observers.

	tarsus length				body condition				wing length				tail length			
**fixed effects**	est.	SE	*t*	*P*	est.	SE	*t*	*P*	est.	SE	*t*	*P*	est.	SE	*t*	*P*
intercept	19.95	0.10	195.38	<2.E-16	4.15	1.31	3.17	1.6.E-3	60.60	2.82	21.50	<2.E-16	56.15	4.24	13.24	<2.E-16
habitat-urban	-0.18	0.12	-1.54	0.13	-0.12	0.20	-0.58	0.56	-0.34	0.51	-0.67	0.50	-0.43	0.74	-0.58	0.56
year-2014	0.06	0.08	0.72	0.48	-0.81	0.20	-4.01	7.E-05	-0.95	0.55	-1.72	0.09	-0.03	0.74	-0.04	0.97
year-2015	0.01	0.09	0.17	0.87	-1.21	0.22	-5.58	4.E-08	0.42	0.58	0.72	0.47	1.71	0.78	2.20	0.03
year-2016	-0.04	0.10	-0.39	0.70	-0.88	0.22	-3.97	8.E-05	-0.57	0.56	-1.01	0.31	1.42	0.79	1.80	0.07
year-2017	0.19	0.13	1.48	0.14	-0.33	0.23	-1.45	0.147	0.29	0.54	0.54	0.59	1.02	0.80	1.28	0.20
sex-fem	-0.56	0.12	-4.59	6.E-06	-0.57	0.21	-2.73	0.01	-2.73	0.46	-5.94	6.E-09	-3.62	0.72	-5.06	6.E-07
age-yearling	0.04	0.06	0.61	0.54	-0.30	0.15	-2.03	0.04	-1.51	0.34	-4.46	1.E-5	-2.13	0.50	-4.28	0.00
tarsus length	-	-	-	-	0.66	0.06	10.34	<2.E-16	0.83	0.14	6.02	3.9.E-9	0.45	0.21	2.15	0.03
hbt-urb×age-yrl	-0.05	0.07	-0.65	0.51	-0.14	0.15	-0.92	0.36	-0.66	0.36	-1.86	0.06	-0.04	0.51	-0.09	0.93
hbt-urb×sex-fem	-0.12	0.18	-0.70	0.48	-0.13	0.16	-0.83	0.41	-0.04	0.33	-0.11	0.91	0.29	0.52	0.55	0.58
sex-fem×age-yrl	-0.09	0.06	-1.47	0.14	0.15	0.14	1.09	0.28	0.84	0.31	2.70	0.01	0.65	0.44	1.47	0.14
year-14×sex-Fem	0.03	0.08	0.42	0.68	0.38	0.21	1.86	0.06	-0.02	0.50	-0.04	0.97	0.13	0.70	0.19	0.85
year-15×sex-Fem	0.09	0.09	1.01	0.31	0.35	0.21	1.69	0.09	0.76	0.50	1.53	0.13	-0.30	0.70	-0.43	0.67
year-16×sex-Fem	0.19	0.09	2.07	0.04	0.22	0.20	1.11	0.27	-0.05	0.47	-0.10	0.92	0.17	0.67	0.25	0.81
year-17×sex-Fem	0.04	0.10	0.37	0.71	0.23	0.21	1.11	0.27	-0.22	0.48	-0.46	0.65	-0.01	0.69	-0.02	0.98
hbt-urb×year-14	0.03	0.09	0.38	0.70	-0.14	0.22	-0.65	0.51	0.24	0.58	0.42	0.67	-0.33	0.78	-0.42	0.68
hbt-urb×year-15	-0.13	0.10	-1.34	0.18	0.34	0.23	1.45	0.15	-1.32	0.61	-2.16	0.03	-1.36	0.83	-1.65	0.10
hbt-urb×year-16	-0.08	0.11	-0.77	0.44	0.05	0.23	0.21	0.84	0.36	0.57	0.63	0.53	-0.55	0.82	-0.68	0.50
hbt-urb×year-17	-0.28	0.13	-2.09	0.04	-0.20	0.24	-0.85	0.39	-0.28	0.58	-0.48	0.63	-0.46	0.83	-0.56	0.58
hour	-	-	-	-	9.E-4	3.E-4	3.14	1.8.E-3	-	-	-	-	-	-	-	-
**random effects**	variance		sample size		variance		sample size		variance		sample size		variance		sample size	
individual ring ID	0.28		nb indiv. = 449		3.E-01		nb indiv. = 446		0.14		nb indiv. = 449		2.71		nb indiv. = 441	
observer	0.00		nb obs. = 13		4.E-46		nb obs. = 13		0.15		nb obs. = 13		0.00		nb obs.= 12	
residuals	0.02		N = 554		3.E-01		N = 550		2.78		N =553		3.39		N = 537	

Table S3: Comparing the morphological traits tarsus length, relative body mass, relative wing length and relative tail length between forest and urban great tits using linear mixed models with individual identity and observer identity as random effects. Tarsus, wing and tail lengths are expressed in millimeters and body condition in grams. Abbreviations: fem = female, hbt = habitat, yrl = yearling, nb indiv. = number of individuals, nb obs. = number of observers.

	**tarsus length**				**body mass**				**wing length**				**tail length**			
**mean ± SD**	forest		city		forest		city		forest		city		forest		city	
females	19.54 ± 0.51		19.23 ± 0.53		16.61 ± 0.88		16.20 ± 0.89		73.39 ± 1.80		72.58 ± 1.70		61.24 ± 2.55		60.87 ± 2.43	
males	20.01 ± 0.51		19.76 ± 0.57		17.25 ± 0.94		16.98 ± 0.97		76.43 ± 2.09		75.59 ± 2.29		65.35 ± 2.84		64.37 ± 2.93	
**fixed effects**	est.	SE	*t*	*P*	est.	SE	*t*	*P*	est.	SE	*t*	*P*	est.	SE	*t*	*P*
intercept	19.99	0.06	357.70	**<2E-16**	4.03	1.29	3.12	**2.E-03**	60.12	2.81	21.37	**<2e-16**	56.77	4.15	13.69	**<2e-16**
habitat-urban	-0.27	0.06	-4.73	**3.E-06**	-0.13	0.10	-1.26	0.21	-0.64	0.48	-1.34	0.18	-0.91	0.26	-3.50	**6.E-04**
year-2014	ns	ns	ns	ns	-0.69	0.10	-6.69	**7.E-11**	-0.87	0.50	-1.76	0.08	-0.24	0.35	-0.69	0.50
year-2015	ns	ns	ns	ns	-0.78	0.10	-7.49	**3.E-13**	0.64	0.53	1.22	0.23	0.59	0.35	1.67	0.10
year-2016	ns	ns	ns	ns	-0.71	0.10	-7.08	**5.E-12**	-0.71	0.51	-1.39	0.17	1.05	0.34	3.12	**3.E-03**
year-2017	ns	ns	ns	ns	-0.37	0.10	-3.55	**4.E-04**	0.28	0.49	0.58	0.57	0.66	0.35	1.90	**0.07**
sex-fem	-0.49	0.05	-9.73	**<2E-16**	-0.37	0.08	-4.67	**4.E-06**	-2.60	0.20	-12.88	**<2E-16**	-3.15	0.25	-12.59	**<2e-16**
age-yearling	ns	ns	ns	ns	-0.08	0.12	-0.71	0.48	-1.94	0.23	-8.53	**<2E-16**	-1.87	0.22	-8.58	**<2e-16**
tarsus length	ns	ns	ns	ns	0.66	0.06	10.39	**<2E-16**	0.87	0.14	6.26	**1.E-09**	0.43	0.21	2.09	**0.04**
hbt-urb×year-14	ns	ns	ns	ns	-0.16	0.22	-0.73	0.47	0.14	0.58	0.25	0.81	ns	ns	ns	ns
hbt-urb×year-15	ns	ns	ns	ns	0.38	0.23	1.68	0.09	-1.13	0.61	-1.85	0.07	ns	ns	ns	ns
hbt-urb×year-16	ns	ns	ns	ns	0.86	0.23	0.38	0.70	0.47	0.57	0.81	0.42	ns	ns	ns	ns
hbt-urb×year-17	ns	ns	ns	ns	0.19	0.24	-0.82	0.42	-0.35	0.58	-0.59	0.55	ns	ns	ns	ns
hbt-urb×age-yrl	ns	ns	ns	ns	-0.28	0.14	-1.99	0.05	ns	ns	ns	ns	ns	ns	ns	ns
sex-fem×age-yrl	ns	ns	ns	ns	ns	ns	ns	ns	0.77	0.31	2.51	**0.01**	ns	ns	ns	ns
hour	-	-	-	-	8.E-04	0.00	2.91	**4.E-03**	-	-	-	-	-	-	-	-
**random effects**	variance		sample size		variance		sample size		variance		sample size		variance		sample size	
individual ring ID	0.28		nb indiv. = 449		0.30		nb indiv. = 446		0.26		nb indiv. = 449		2.87		nb indiv. = 441	
observer	0.00		nb obs. = 13		0.01		nb obs. = 13		0.18		nb obs. = 13		0.01		nb obs.= 12	
residuals	0.02		N = 554		0.30		N = 550		2.67		N =553		3.22		N = 537	

Table S4: Output of complete initial linear models (LMER) on life-history traits before removing non-significant variables. Abbreviations: hbt-urb = urban habitat (contrasted with forest habitat), nb female ID = number of females.

	laying date				clutch size			
**fixed effects**	est.	SE	*t*	*P*	est.	SE	*t*	*P*
intercept	99.00	1.73	57.19	<2.E-16	11.20	0.90	12.41	<2.E-16
habitat-urban	-4.05	1.89	-2.15	0.032	-1.60	0.42	-3.85	1.E-04
year-2012	7.96	2.29	3.48	5.E-04	-0.59	0.51	-1.15	0.25
year-2013	10.05	2.32	4.33	1.69E-05	0.64	0.53	1.20	0.23
year-2014	-4.06	2.08	-1.95	5.E-02	-0.07	0.46	-0.15	0.88
year-2015	6.10	2.13	2.87	4.E-03	-0.74	0.48	-1.53	0.13
year-2016	1.45	2.12	0.69	0.49	-0.20	0.47	-0.43	0.67
year-2017	-5.37	2.03	-2.64	8.E-03	-0.46	0.45	-1.03	0.31
hbt-urb×year-12	-2.35	2.53	-0.93	0.35	-0.21	0.56	-0.37	0.71
hbt-urb×year-13	-2.92	2.58	-1.13	0.26	-0.77	0.58	-1.32	0.19
hbt-urb×year-14	1.49	2.35	0.64	0.53	-0.27	0.52	-0.51	0.61
hbt-urb×year-15	-0.40	2.41	-0.17	0.87	0.03	0.54	0.06	0.95
hbt-urb×year-16	2.23	2.38	0.94	0.35	-0.17	0.53	-0.32	0.75
hbt-urb×year-17	3.83	2.27	1.69	0.09	0.09	0.50	0.19	0.85
laying date	-	-	-	-	-0.02	0.01	-2.16	0.03
**random effects**	variance		sample size		variance		sample size	
female ID	19.39		nb female ID = 662		0.66		nb female ID = 651	
residuals	28.57		N = 724		1.66		N = 713	

Table S5: Comparing laying date and clutch size between forest and urban great tits using linear mixed models with female individual identity as random effect. Abbreviations: hbt-urb = urban habitat (contrasted with forest habitat), nb female ID = number of females.

	laying date				clutch size			
**mean ± SD**	forest		city		forest		city	
	100.01 ± 7.24 *		97.2 ± 8.35 *		9.16 ± 1.75		7.46 ± 1.51	
**fixed effects**	est.	SE	*t*	*P*	est.	SE	*t*	*P*
intercept	99.00	1.73	57.19	**<2.E-16**	11.23	0.83	13.54	**<2.E-16**
habitat-urban	-4.05	1.89	-2.15	**0.032**	-1.75	0.14	-12.61	**<2.E-16**
year-2012	7.96	2.29	3.48	**5.E-04**	-0.76	0.22	-3.45	**6.E-04**
year-2013	10.05	2.32	4.33	**2.E-05**	0.01	0.23	0.04	0.97
year-2014	-4.06	2.07	-1.95	0.051	-0.27	0.22	-1.26	0.21
year-2015	6.10	2.12	2.87	**0.004**	-0.74	0.23	-3.25	**1.E-03**
year-2016	1.45	2.12	0.69	0.493	-0.34	0.22	-1.57	0.12
year-2017	-5.37	2.03	-2.64	**0.008**	-0.41	0.20	-2.04	**4.E-02**
hbt-urb×year-12	-2.35	2.53	-0.93	0.354	ns	ns	ns	ns
hbt-urb×year-13	-2.92	2.58	-1.13	0.258	ns	ns	ns	ns
hbt-urb×year-14	1.49	2.35	0.64	0.526	ns	ns	ns	ns
hbt-urb×year-15	-0.40	2.41	-0.17	0.869	ns	ns	ns	ns
hbt-urb×year-16	2.23	2.38	0.94	0.350	ns	ns	ns	ns
hbt-urb×year-17	3.83	2.27	1.69	0.092	ns	ns	ns	ns
laying date	-	-	-	-	-0.02	0.01	-2.05	**4.E-02**
**random effects**	variance		sample size		variance		sample size	
female ID	19.38		nb female ID = 662		0.74		nb female ID = 651	
residuals	28.57		N = 724		1.59		N = 713	

* Laying date is expressed in ordinal day, where 1 = January the 1st.

Table S6: Standardised linear and quadratic selection differentials on tarsus length, body condition, wing length and tail length. Values are provided with their standard errors. Bold estimates are significant (P<0.05). “int.” displays the value of the coefficient for interaction between trait and habitat in the merged (forest + city) model.

selection differentials	sex	habitat	tarsus					body mass					wing					tail				
			N	est.	±	SE	int.	N	est.	±	SE	int.	N	est.	±	SE	int.	N	est.	±	SE	int.
linear	female	forest	110	-0.001	±	0.06	-0.023	109	-0.036	±	0.06	-0.013	110	-0.007	±	0.06	0.019	105	-0.085	±	0.061	0.06
		city	202	-0.022	±	0.043		166	-0.022	±	0.044		202	0.062	±	0.043		201	-0.004	±	0.044	
	male	forest	105	0.075	±	0.059	-0.073	102	-0.086	±	0.06	**0.148**	104	0.003	±	0.06	0.052	96	0.042	±	0.061	-0.003
		city	195	0.008	±	0.042		159	0.05	±	0.042		195	0.054	±	0.042		192	0.036	±	0.042	
quadratic	female	forest	110	0.032	±	0.081	-2.29	109	-0.067	±	0.071	0.04	110	0.109	±	0.066	-0.106	105	0.107	±	0.074	-0.038
		city	202	-0.082	±	0.054		166	0.039	±	0.05		202	-0.016	±	0.054		201	0.035	±	0.059	
	male	forest	105	0.178	±	0.09	-3.506	102	-0.025	±	0.066	0.056	104	0.057	±	0.089	-0.122	96	-0.04	±	0.93	0.026
		city	195	-0.008	±	0.058		159	0.037	±	0.068		195	-0.031	±	0.065		192	-0.031	±	0.06	

Table S7: Standardised quadratic selection gradients on tarsus length, body mass, wing length and tail length. Values are provided with their standard errors. The bold estimate is significant (P=0.036). "Int." displays the value of the coefficient for interaction between trait and habitat in the merged (forest + city) model.

sex	habitat	sample size	quadratic selection gradients															
			γ_{Tarsus}				$\gamma_{Body\ mass}$				γ_{Wing}				γ_{Tail}			
			est.	±	SE	Int.	est.	±	SE	Int.	est.	±	SE	Int.	est.	±	SE	Int.
female	forest	104	-0.018	±	0.130	-0.043	-0.076	±	0.090	0.021	0.002	±	0.136	-0.017	0.161	±	0.114	-0.011
	city	200	**-0.166**	**±**	**0.076**		-0.121	±	0.070		-0.142	±	0.080		0.036	±	0.070	
male	forest	94	0.192	±	0.112	-0.095	0.042	±	0.096	0.020	0.094	±	0.112	-0.048	0.050	±	0.138	-0.009
	city	191	-0.006	±	0.088		0.080	±	0.120		-0.220	±	0.164		-0.146	±	0.106	

Table S8: Comparison of variances between urban and forest habitat for morphological and life history traits. Fisher variance ratio test (*var.test()*, r package *stats*). d.f. = degrees of freedom, c.i = 95% confidence interval of f.

	**Urban variance**	**Forest variance**	**F-test**			
			d.f.	c.i.	F	*P*
**tarsus length**	0.38	0.32	171	0.65 - 1.08	0.83	0.17
**body mass**	1.02	0.93	169	0.71 - 1.20	0.91	0.5
**wing length**	6.3	6.1	170	0.75 - 1.26	0.97	0.811
**tail length**	10.28	11.44	158	0.86 - 1.46	1.11	0.41
**laying date**	69.67	52.4	190	0.60 - 0.96	0.75	**0.02**
**clutch size**	2.27	3.07	184	1.07 - 1.73	1.35	**0.01**

Table S9: Standardised linear and quadratic selection differentials on laying date and clutch size. Bold estimates are significant (P<0.05). "Interaction" displays the value of the coefficient for interaction between trait and habitat in the merged (forest + city) model.

selection differentials	habitat	laying date					clutch size				
		N	est.	±	SE	interaction	N	est.	±	SE	interaction
**linear**	**forest**	191	**-0.163**	**±**	**0.056**	**0.318**	185	0.088	±	0.057	**0.118**
	**city**	527	0.073	±	0.032		533	**0.205**	**±**	**0.031**	
**quadratic**	**forest**	191	-0.017	±	0.087	0.012	185	**-0.122**	**±**	**0.062**	0.102
	**city**	527	-0.019	±	0.043		533	-0.028	±	0.038	

Table S10: Standardised quadratic selection gradients on laying date and clutch size. Values are provided with their standard errors. The underlined estimate is marginally significant (P=0.054). "Interaction" displays the value of the coefficient for interaction between trait and habitat in the merged (forest + city) model.

habitat	sample size	quadratic selection gradients							
		$\gamma_{Laying\ date}$				$\gamma_{Clutch\ size}$			
		est.	±	SE	interaction	est.	±	SE	interaction
forest	94	-0.004	±	0.089	-0.006	-0.115	±	0.062	0.100
city	191	4.E-04	±	0.042		-0.028	±	0.038	

Supplementary references:

Gosler AG, Harper DGC. 2000 Assessing the heritability of body condition in birds: a challenge exemplified by the great tit *Parus major* L. (Aves), *Biol. J. Linn. Soc.* **71**, 103–117. (doi:10.1111/j.1095-8312.2000.tb01245.x)

Naef-Daenzer B. 2000 Patch time allocation and patch sampling by foraging great and blue tits. *Animal behaviour*, **59**, 989-999. (doi:10.1006/anbe.1999.1380)

QGIS Development Team 2018. QGIS Geographic Information System. Open source geospatial foundation project. http://qgis.osgeo.org

Postma E. 2014 Four decades of estimating heritabilities in wild vertebrate populations: improved methods, more data, better estimates. *Quantitative genetics in the wild*, 16-33.
